# Surveillance burden in pulmonary nodule management

**DOI:** 10.3389/fmed.2026.1971481

**Published:** 2026-09-08

**Authors:** Huijuan Cheng, Zhe Zhang, Yanyan Ye, Feng Lu

**Affiliations:** 1 Department of Respiratory Medicine, The Second Affiliated Hospital of Fujian University of Traditional Chinese Medicine, Fuzhou, China; 2 Fujian University of Traditional Chinese Medicine, Fuzhou, China

**Keywords:** computed tomography, guideline adherence, lung cancer, oncologic risk, patient-centered care, pulmonary nodule, surveillance burden

## Introduction

Computed tomography (CT) surveillance is central to managing indeterminate pulmonary nodules when malignancy probability does not warrant immediate invasive evaluation or treatment (1–3). Although surveillance avoids premature procedures, it remains an active longitudinal care process: prior examinations must be retrieved and compared, follow-up imaging scheduled and completed, results communicated, and subsequent decisions revisited under uncertainty.

We propose surveillance burden as the cumulative workload, exposures, and patient-experienced consequences generated by longitudinal nodule follow-up, assessed together with care-process barriers that may create or amplify them. Drawing on Burden of Treatment Theory, which links health-care workload with patients' capacity to respond (4), we present it as a multilevel, hypothesis-generating framework rather than a validated clinical construct, syndrome, score, or decision rule. Its proposed advance is not to relabel anxiety, access problems, or imaging overuse; it is to place these established concerns within a systematic implementation layer that complements, but does not override, guideline-based oncologic risk assessment. This Opinion synthesizes published evidence and presents no original or unpublished patient data.

### What surveillance burden adds

As proposed here, surveillance burden is intended to complement rather than duplicate patient-centered care, shared decision-making, care coordination, adherence interventions, or guideline implementation. These approaches serve different functions: patient-centered care and shared decision-making address goals, preferences, and deliberation, whereas coordination, adherence, and implementation strategies focus on organizing or completing recommended care. The proposed framework instead uses the longitudinal surveillance pathway as a common analytic frame linking objective workload and exposure, patient-reported consequences, and system-process barriers over time. Joint consideration may reveal cross-domain cascades and trade-offs that single-domain approaches may miss. For example, retrieving prior imaging can simultaneously prevent duplicate CT, reduce radiation and logistical workload, lessen uncertainty, and improve continuity. Its hypothesized incremental value lies in integration, providing a common structure for measuring interacting consequences and testing coordinated implementation strategies while the oncologic pathway remains unchanged.

Evidence supports the relevance of individual components, although not yet their integration as one construct. Patients have described uncertainty as a major consequence of indeterminate nodules (5). In the Watch the Spot Trial, emotional distress and anxiety varied across patients, and aspects of communication were associated with psychological outcomes (6). Among 936 participants with screening-detected nodules, clinically significant anxiety, depression, and insomnia were reported in 40.2%, 31.7%, and 24.6% at baseline, respectively (7). A systematic review also identified tracking systems, standardized reporting, decision support, process improvement, and patient involvement as strategies for improving guideline-concordant follow-up (8).

The evidence also shows that both under-surveillance and over-surveillance can be harmful. Follow-up completion is inconsistent (8, 9), whereas 37.2% of patients in one cohort underwent CT more frequently than recommended (10). In the Watch the Spot radiation analysis, cumulative 2-year effective dose did not differ materially between trial arms; however, per-examination doses varied across sites, and most screening and follow-up examinations exceeded the size-adjusted American College of Radiology/American Association of Physicists in Medicine thresholds evaluated in that study (11). Appropriate surveillance therefore means risk-concordant care: it may be more intensive when oncologic risk warrants escalation and less intensive only when the applicable guideline permits it.

For hypothesis generation, we provisionally organize five domains by their primary mechanism: (i) imaging/radiation exposure (number, timing, duration, and cumulative dose of CT examinations); (ii) psychological impact (cancer worry, uncertainty, anxiety, and sleep disturbance); (iii) logistical burden (travel, time, financial cost, work disruption, and access); (iv) clinical/diagnostic burden (incidental findings, additional tests or referrals, and interaction with comorbidity or pulmonary reserve); and (v) system-level barriers (fragmented care, inaccessible prior imaging, incomplete communication, loss to follow-up, and unclear ownership). These domains are deliberately not assumed to be mutually exclusive. For example, failed communication is recorded as a system-level event and its anxiety-related consequence as a patient-reported outcome. Future studies should preserve such indicators at their measurement level rather than double-counting them in an additive score. Thus, the proposed framework spans patient-reported burden, objective workload or exposure, and care-process conditions; it is not limited to subjective experience.

### Oncologic risk sets the safety floor

**Figure 1:**
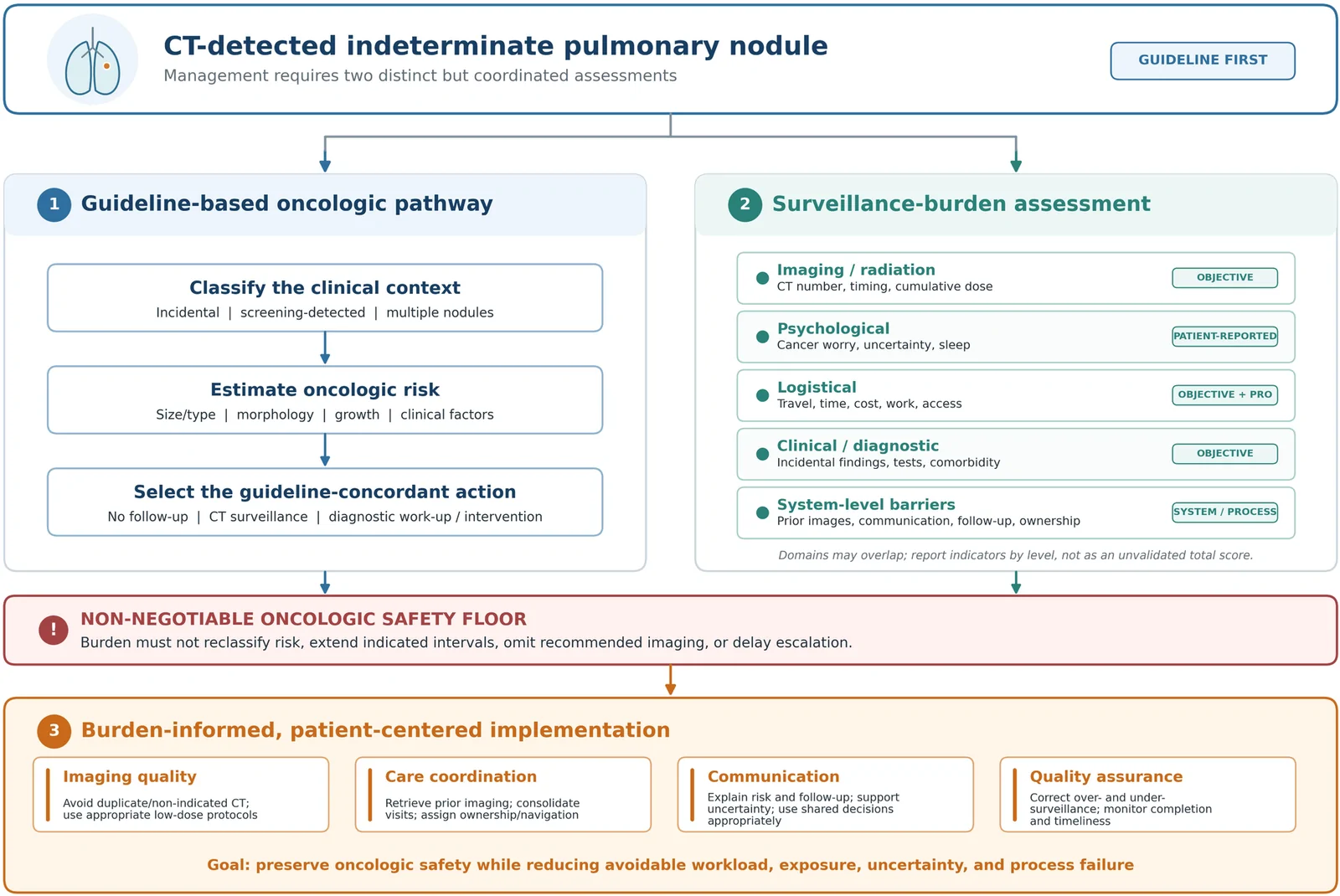
Proposed hypothesis-generating framework for guideline-first pulmonary nodule surveillance. This conceptual model is not a validated clinical construct or decision algorithm. After the clinical context is classified, oncologic risk determines the guideline-concordant pathway and establishes a non-negotiable safety floor. Within the proposed framework, surveillance burden is provisionally organized across five domains using candidate objective, patient-reported, and system-process indicators. Burden-informed implementation is a testable proposition that may improve imaging quality, care coordination, communication, and quality assurance; however, any future clinical use must not reclassify risk, extend indicated intervals, omit recommended imaging, or delay diagnostic escalation. Because domains may overlap, their indicators should initially be reported separately rather than combined into a score before validation.

Figure 1 illustrates the proposed hypothesis-generating framework, which conceptually separates two operations that should not be conflated. First, clinicians classify the nodule and select a management pathway using the applicable oncologic framework. Fleischner recommendations apply to incidentally detected nodules within their defined population (1), whereas screen-detected nodules follow Lung-RADS or the relevant screening protocol (3). For multiple nodules, the applicable guidance requires patient- and lesion-level assessment rather than assuming that every lesion carries the same risk (1, 3). Second, the proposed framework organizes burden-related factors that may inform future efforts to optimize implementation of the selected pathway. This distinction also defines scope: the proposal concerns adults with CT-detected indeterminate nodules managed longitudinally, whether found incidentally or through screening, while recognizing that structured screening programs, incidental discovery across care settings, and multiple-nodule surveillance may produce different burden profiles.

Oncologic risk therefore establishes a non-negotiable safety floor. Any future clinical use of burden assessment, if validated, must not reclassify risk, extend an indicated interval, omit recommended imaging, or delay diagnostic escalation. Adaptable elements are those concerning delivery: eliminating duplicate or non-indicated CT, retrieving prior images, using standardized low-dose protocols when diagnostically appropriate, consolidating visits, assigning clear ownership and navigation, improving risk communication, offering psychological support, and correcting excessive follow-up. By contrast, guideline-defined size or risk categories, indicated follow-up intervals, imaging needed to assess growth, and triggers for diagnostic evaluation remain unchanged unless prospective evidence and subsequent guidance support modification.

Any future burden-adapted implementation should remain patient-centered without treating patient-centered decision-making and shared decision-making as synonyms. Patient-centered decision-making is the broader orientation to a person's goals, context, and capacity. Shared decision-making is a specific deliberative process for situations in which more than one guideline-concordant option exists and patient preferences can legitimately influence the choice (2). Neither concept authorizes departure from oncologic safety requirements. Digital tracking and automated longitudinal comparison may reduce some process failures, but technology alone cannot resolve poor communication or unclear responsibility (8).

### A staged research agenda

Measurement should precede scoring. Candidate objective indicators include CT number and timing, cumulative dose, travel time, appointment count, missed visits, and downstream tests. Patient-reported measures should capture cancer worry, uncertainty, sleep disturbance, and perceived time or financial strain. System-process measures should include accessibility of prior imaging, closed-loop communication, loss to follow-up, and explicitly assigned responsibility. These levels should initially be reported separately, because their relative importance, directionality, and potential overlap remain unknown.

A staged research program is required. First, measurement and feasibility studies should establish content validity with patients and clinicians, reliability, and interpretability across incidental, screening-detected, and multiple nodules. Second, prospective observational studies should test—rather than assume—whether domain-specific measures are associated with guideline-concordant completion, unscheduled health-care use, disparities, or preference-sensitive decisions. Third, comparative intervention studies should evaluate burden-informed implementation against usual care while retaining oncologic outcomes, including timely cancer diagnosis, stage, interval growth, invasive procedures, and mortality, alongside radiation, utilization, anxiety, uncertainty, quality of life, time cost, and access.

## Discussion

The evidence cited here supports the importance of individual problems, including psychological distress, incomplete or excessive follow-up, radiation variation, and care-process failures, but it does not clinically validate their integration as surveillance burden. The proposed framework is therefore hypothesis-generating rather than a validated clinical construct. Its incremental conceptual contribution is a common longitudinal structure for examining interactions among patient experience, objective surveillance workload and exposure, and care-process barriers while preserving the primacy of context-specific oncologic guidance. Structured multidisciplinary care may improve patient-centered outcomes in selected multiple-nodule populations, but observational evidence should not be interpreted as validation of this framework (12).

The proposal has important limitations: domain boundaries overlap, measurement properties and weighting are unknown, and burden may differ between incidental and screening pathways and across health systems. Nevertheless, separating oncologic risk assessment from burden-informed implementation clarifies what may be adapted and what must remain protected. Two patients with similar nodules may require the same oncologic schedule yet need very different communication, coordination, and practical support. If validated, the framework could help adapt how risk-based follow-up is delivered without lowering the standard of follow-up.
